# Eye Movements Affect Postural Control in Young and Older Females

**DOI:** 10.3389/fnagi.2016.00216

**Published:** 2016-09-16

**Authors:** Neil M. Thomas, Theodoros M. Bampouras, Tim Donovan, Susan Dewhurst

**Affiliations:** Active Ageing Research Group, Department of Medical and Sport Sciences, University of CumbriaLancaster, UK

**Keywords:** balance, elderly, eye tracking, gaze accuracy, saccadic, smooth pursuit, visual input

## Abstract

Visual information is used for postural stabilization in humans. However, little is known about how eye movements prevalent in everyday life interact with the postural control system in older individuals. Therefore, the present study assessed the effects of stationary gaze fixations, smooth pursuits, and saccadic eye movements, with combinations of absent, fixed and oscillating large-field visual backgrounds to generate different forms of retinal flow, on postural control in healthy young and older females. Participants were presented with computer generated visual stimuli, whilst postural sway and gaze fixations were simultaneously assessed with a force platform and eye tracking equipment, respectively. The results showed that fixed backgrounds and stationary gaze fixations attenuated postural sway. In contrast, oscillating backgrounds and smooth pursuits increased postural sway. There were no differences regarding saccades. There were also no differences in postural sway or gaze errors between age groups in any visual condition. The stabilizing effect of the fixed visual stimuli show how retinal flow and extraocular factors guide postural adjustments. The destabilizing effect of oscillating visual backgrounds and smooth pursuits may be related to more challenging conditions for determining body shifts from retinal flow, and more complex extraocular signals, respectively. Because the older participants matched the young group's performance in all conditions, decreases of posture and gaze control during stance may not be a direct consequence of healthy aging. Further research examining extraocular and retinal mechanisms of balance control and the effects of eye movements, during locomotion, is needed to better inform fall prevention interventions.

## 1. Introduction

Vision is an important sensory cue to familiarize ourselves with the external environment, a prerequisite for which are voluntary or involuntary eye movements, necessary to process information such as recognition, localization and proprioception (Irwin, 1991; Lewis et al., 1994; Donaldson, 2000). Vision also facilitates stabilization of upright posture, by enabling detection of self-motion relative to structures in the visual field (Dichgans and Brandt, 1978). There is growing evidence to suggest eye movements interact with this process (Schulmann et al., 1987; Glasauer et al., 2005; Guerraz and Bronstein, 2008; Laurens et al., 2010; Rodrigues et al., 2015). However, this has received little attention in the gerontology literature, which is surprising given the prevalence of eye movements in everyday life (Kowler, 2011), their potential link with postural control, and the high incidence of falls and fall related injuries amongst the elderly (Sturnieks et al., 2008; Ambrose et al., 2013). Here our focus is on the effects of eye movements on postural control in young and older individuals.

Visual cues for postural stabilization have traditionally been associated with deformation of the retinal image. As a person shifts their position in space, changes in the pattern of light intensities about a central point of observation create an optic flow pattern, which is projected onto the retina. This projected image shifts/deforms creating retinal flow according to an individual's movements (Gibson, 1950), which the central nervous system (CNS) uses to estimate body position and initiate appropriate postural adjustments (Wapner and Witkin, 1950; Lestienne et al., 1977; Nashner and Berthoz, 1978). Optical changes at the retina can include uniform components (e.g., horizontal movement of the retinal image), parallax (generated by near and far structures in the visual environment), and expansion and contraction (indicative of anterior or posterior head motion; Gibson, 1950; Gibson et al., 1955). Evidence demonstrating how retinal flow guides postural adjustments can be taken from investigations involving moving visual surrounds, e.g., linearly oscillating walls, floors and tunnels, which have frequently shown a coupling of body sway with stimulus motion (Lee and Lishman, 1975; Stoffregen, 1985; Bronstein, 1986; Stoffregen, 1986; Flückiger and Baumberger, 1988; Dijkstra et al., 1994). This is believed to result from the CNS misinterpreting external-motion for self-motion and incorrectly adjusting body orientation (Guerraz and Bronstein, 2008).

There is a close relationship between the ways in which visual and vestibular information about head position are used for postural control (DeAngelis and Angelaki, 2012), and eye movements have been shown to affect posture during standing (Paulus et al., 1984). Fixating on a small lit target in an otherwise dark room improved stability compared to absolute dark (Paulus et al., 1984). In these conditions, visual and vestibular initiated compensatory eye movements in response to movements of the head keep gaze fixated on the target, implying diminished retinal flow. Therefore, eye movements relative to the target are used to infer body position in space (extraocular balance control; Guerraz and Bronstein, 2008). Visually tracking moving targets (smooth pursuits) caused increases of postural sway in young adults, in the presence of a static visual field and without (Glasauer et al., 2005; Laurens et al., 2010). This may be related to more challenging conditions for interpreting retinal flow for postural control (Schulmann et al., 1987), or, in part, more complex extraocular signals (Laurens et al., 2010). However, there are data which show an opposite effect, indicating posture can be modulated for more accurate gaze behavior (Rodrigues et al., 2015). This concurs with similar findings during rapid shifts of gaze from one target to another (saccades) in young (Stoffregen et al., 2006; Rougier and Garin, 2007; Stoffregen et al., 2007; Rodrigues et al., 2013, 2015) and older (Aguiar et al., 2015) adults, suggesting a functional integration of gaze and posture for both smooth pursuit and saccadic eye movements. These differences remain unexplained. Moreover, little is known about extraocular control of posture in elders, or how smooth pursuits effect balance in elders.

Older individuals have demonstrated declines in visual self-motion perception (Warren et al., 1989), and can become more unstable in the face of moving visual surrounds (Wade et al., 1995; Sundermier et al., 1996; Borger et al., 1998). This might reduce their ability to interpret retinal flow for postural control as effectively as younger adults during eye movements. Declines in vestibulo-ocular reflex (VOR) function with age (Peterka et al., 1990; Paige, 1991; Baloh et al., 2003) may additionally affect the extraocular component of postural control, since the VOR is one mechanism which serves to stabilize gaze, and eye movement signals appear to be used to infer body position. Further, an inaccurate smooth pursuit system in elders (Sharpe and Sylvester, 1978; Spooner et al., 1980; Moschner and Baloh, 1994; Ross et al., 1999; Knox et al., 2005) may potentially cause less efficient processing of more complex extraocular signals whilst visually tracking moving targets, exacerbating the increase in postural sway demonstrated by some young adults. Paquette and Fung (2011) indirectly assessed balance during smooth pursuits in older participants, but the authors focus was gaze accuracy, and they cannot clarify if declines in postural control were associated with the gaze outcomes.

Because loss of balance in the elderly can be costly and debilitating (Brunner et al., 2003), there is a pressing need to further understanding of the interplay between eye movements and postural control in this population. Therefore, our aim was to assess postural sway, increases of which can indicate increased risk of falls, during visual fixation of stationary targets, smooth pursuits and saccades, in young and older individuals. We also used combinations of absent, fixed, and horizontally oscillating visual backgrounds to generate different forms of retinal flow and to isolate the extraocular factors involved in visual control of balance. Finally, we assessed accuracy of gaze to determine if different backgrounds altered gaze behavior, and to examine differences in error rates between age groups. We hypothesized: (1) fixating a stable target to reduce body sway; (2) fixed backgrounds to have a stabilizing effect and oscillating backgrounds to have a destabilizing effect; (3) smooth pursuits to increase body sway; (4) saccades to decrease body sway; (5) elders to be more unstable throughout, with greater effects during smooth pursuits and oscillating backgrounds; (6) gaze accuracy to decline in the older group.

## 2. Materials and methods

### 2.1. Participants

Twelve young (mean ± SD: age: 26.1 ± 4.9 years, height: 1.68 ± 0.06 m, mass: 62.2 ± 13.7 kg) and 12 older (mean ± SD: age: 72.8 ± 6.9 years, height: 1.64 ± 0.05 m, mass: 63.6 ± 10.7 kg) females participated in the study. The older participants were interviewed by telephone to determine suitability. An initial cohort of 20 elders was reduced to 12 following screening by self-report for the following inclusion criteria: (1) No macular degeneration, glaucoma, cataracts or color blindness; (2) No muscle or bone conditions which could prevent standing for 30 min with breaks including (but not limited to) lower limb, hip or spine surgery within the previous year, present of recent injury or pain in any region which could arise from standing; (3) No psychological/neurological conditions which could prevent observation of a visual scene or standing for 30 min with breaks including (but not limited to) Parkinsons disease, vestibular impairment (dizziness/vertigo), numbness or loss of sensation in the lower limbs, or schizophrenia; (4) No severe motion sickness; (5) No medication which could depress the nervous system or effect balance (benzodiazepines, anti-depressants, anti-seizure, or anti-anxiety); (6) No multiple falls within the previous year; (7) No over-reliance on handrails when climbing the stairs; (8) No assistive walking devices (cane, crutches, or walking frame). Further, each older participant's mental state was examined with the mini mental status examination, and all achieved a score of ≥24, considered as a minimum acceptable threshold for involvement in the study. The investigation was carried out in accordance with the recommendations of the University of Cumbria's ethical principles and guidelines for research involving human subjects, and all procedures, information to the participants, and participant consent forms, were approved by the University of Cumbria Research Committee. All subjects gave written informed consent in accordance with the Declaration of Helsinki.

### 2.2. Equipment

Visual scenes were rear projected (Sanyo PLC-XU74, Tokyo, Japan) onto a 3.2 × 2.4 m translucent screen. The lower border of the screen was placed at foot level. An AMTI AccuPower portable force platform (AMTI Force and Motion, Watertown, MA, USA) was positioned with its center 1 m adjacent to the middle of the screen. Participants wore eye tracking glasses (Tobii Glasses 2 Eye Tracker, Tobii Technology, Danderyd, Sweden) which have a one point calibration procedure, autoparallax compensation and slippage compensation allowing for persistent calibration throughout testing with no loss of data aside from blinking. The experiment was carried out in a light-controlled room.

### 2.3. Visual scenes

Ten 45 s visual scenes were programmed with Psychopy open-source psychology software (Peirce, 2007). Visual stimuli included a red target (circle with its diameter equivalent to 3° of visual angle) and a large-field background (occupying the full width and height of the screen, made up of black and white vertical stripes each with a width equivalent to 3° of visual angle). Participants had an uncorrected visual acuity ≥20/100 measured on the day of testing. Discrimination of spatial patterns separated by a visual angle of 50/60th of 1° is possible even at lower visual acuities (Paquette and Fung, 2011). Therefore, stimuli utilized in the present investigation were visible at all times, always confirmed with the participant.

The target could be fixed (F), moving smoothly (P) or moving with saccadic motion (S). When fixed, the target would remain in the center of the screen at natural gaze height (see below). When moving smoothly, the target would displace from the center of the screen to 6° of visual angle on the vertical, horizontal or diagonal axis before returning to the center of the screen with a frequency of 0.33 Hz. For saccadic movement the same protocol was implemented, however, the target would disappear from the center of the screen and reappear at the 6° threshold, and vice versa. Target direction was programmed to be random on each oscillation. The large-field background could be absent (N), fixed (F) or oscillating horizontally (6° from the center position in each left and right direction) at 0.33 Hz (O). To simulate a condition of darkness (D) a black screen was projected absent of any stimuli. Letter codes used to identify visual conditions are presented in Table 1. Six degrees of visual angle was chosen to prevent head rotations which could affect measures of body sway, since gaze shifts of >15° are commonly are achieved without rotation of the head (Hallet, 1986), and this method has previously been effective in minimizing head movement (Glasauer et al., 2005; Stoffregen et al., 2006, 2007). We also initiated target movement randomly on the vertical, horizontal and diagonal planes to minimize any systematic bias on one particular axis.

**Table 1:** **Letter codes denoting combinations of large-field background and target state used to identify visual conditions**.

	**Target**		
**Large-field background**	**Fixed**	**Smooth pursuit**	**Saccadic**
None	NF	NP	NS
Fixed	FF	FP	FS
Oscillating	OF	OP	OS
No large-field background or target: Dark (D)			

The first letters refer to the state of the background and second refer to the state of the visual target. Adapted from Laurens et al. (2010).

We used a novel approach regarding the height at which the visual targets were presented, as opposed to eye level. Elders have been shown to adopt forward trunk lean, which may be related to factors such as backward disequilibrium (Manckoundia et al., 2007) or poor balance and fear of falling (Sato and Maitland, 2008). Previous research has also shown focusing gaze at different heights affects measures of postural sway, e.g., 25° up or down from eye level decreased sway velocity and amplitude (Ustinova and Perkins, 2011). Consequently, if the targets were presented at eye level it may have forced the older participants to adopt an unnatural body lean and/or gaze height in order to maintain gaze on the target, which could have affected the results. Therefore, prior to testing, all participants were instructed to stand as still as possible with their feet together (no footwear) in the middle of the force platform (position marked with a cross for accurate relocation between trials) with their hands by their sides. They were then told to look ahead as comfortably as possible at a visual scene consisting of horizontal green lines (full horizontal width of the screen, each covering 2° of visual angle on the vertical plane, and each separated by 2°). After 30 s, gaze fixation settled at a specific line or in between lines. This was considered to be natural gaze height. The participants were subsequently instructed to adopt the same stance position throughout testing, which was reiterated before each trial.

### 2.4. Experimental protocol

Two practice trials of 45 s duration separated by 10–20 s were granted following determination of natural gaze height to familiarize the participants with measurement of postural sway. Following a break of 2–5 min testing commenced. The participants, relocated on the cross and in the same stance as before, were instructed to fixate their gaze on the red target. If the target moved, they should follow it with their eyes only, making sure not to rotate or tilt their head. During the dark condition, they were told to keep looking ahead. The 10 visual scenes were displayed to each participant in a pre-determined random order, different for each participant. After the 3rd and 7th scene the participants were granted a 3–5 min break where they sat down. In between the remaining scenes there was a 10–20 s break where participants remained standing. A member of the research team was present behind each participant during testing in case of loss of balance. The eye tracking glasses were calibrated to each participant before determining natural gaze height, after the practice trials, and subsequently after each 3–5 min break. The calibration procedure adhered to the outlined standardized protocol.

### 2.5. Force platform data

Force platform data were sampled at 100 Hz for 45 s during each trial and analyzed offline (Scipy, Scientific Computing Tools for Python). Since the investigation was not concerned with how quickly the participants adapted to new stimuli, or end anticipation effects, the first and final 5 s were discarded, leaving 35 s of data for analysis (elders have been shown to have similar adaptation rates to young adults regarding sudden changes in visual stimulus motion during an initial 5 s period, Jeka et al., 2010). Medial/lateral (*x*) and anterior/posterior (*y*) center of pressure (COP) coordinate timeseries were then computed and passed through a 4th order zero-lag Butterworth filter with a cut-off frequency of 10 Hz. This choice of cut-off was determined with residual analysis of the raw data (Winter, 1995).

To characterize the size of the path traveled by the COP over the surface of support on both axis, we calculated the root mean square (RMS) of each de-trended timeseries, where *N* = number of data points and *n* = 1, …, *N*:

(1) $$\begin{array}{l} RMS_{x,y}={\left[1/N\sum x,y{\left[n\right]}^{2}\right]}^{1/2} \end{array}$$

Rocchi et al. (2004) recommended RMS to characterize COP coordinate timeseries following principle component analysis. Further, repeated RMS measures of postural sway have been shown to be reliable in young and older populations (Lin et al., 2008).

### 2.6. Gaze fixations

**Figure 1:**
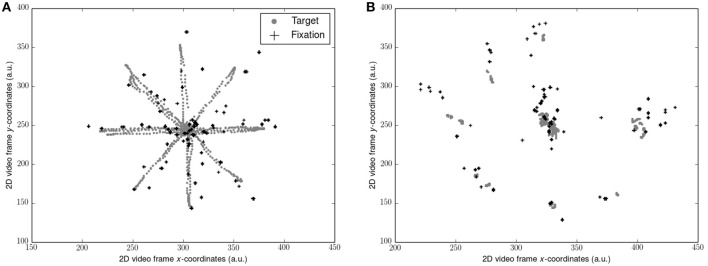
**Representation of target trajectory and gaze fixations from 1 participant: (A) during smooth pursuits; (B) during saccades**. Coordinates along each axis were taken from the 2D video scene relative to the observer and represent arbitrary units (a.u.). Note that the target position is not stable due to the body sway of the observer. Also note the errors of the fixations compared to the target locations.

Gaze data (sampled at 50 Hz) was filtered with the Tobii I-VT fixation filter to yield gaze fixations (window length 20 ms, threshold 30°/s). 2D video sequences consisting of the participants point of view of each visual scene superimposed with their gaze fixations was exported. Position of the target and the position of each gaze fixation as *x* and *y* coordinates on the 2D video frame (Figure 1) was determined using motion tracking software (Open Vision Control). Each video sequence was optically filtered by adapting hue, saturation, brightness and contrast, and luma space level settings in order to improve the accuracy of the tracking algorithm. The resultant coordinate timeseries for each was then calculated where *N* = number of data points and *n* = 1, …, *N*:

(2) $$\begin{array}{l} RC\left[n\right]={\left[x{\left[n\right]}^{2}+y{\left[n\right]}^{2}\right]}^{1/2} \end{array}$$

The first and final 5 s of each timeseries were removed in concordance with the force platform data. Where no gaze data were sampled due to blinking, the target coordinate at the corresponding time point was converted to zero. Errors of gaze relative to the target was then assessed by computing the RMS of gaze subtracted from the target position throughout each video sequence (RMS-gaze error). Reliability of the tracking procedure was assessed by re-tracking the target and fixation position during scene OP from the young participants and computing the coefficient of variation (CV) between the gaze error outcome measures from each track. Scene OP was chosen as it presented with the most challenging optical conditions for motion tracking. The CV between tests (0.47%) indicated excellent reliability. No gaze data were collected for the dark (D) condition.

### 2.7. Statistical analysis

Age (young and older) and condition (10 × visual scenes) were considered as two independent factors. The effects of these two factors on the postural sway outcome variables RMS-*x* and RMS-*y* were examined with a two-way (age × condition) mixed analysis of variance (ANOVA). The effects of the same independent factors minus the dark condition on the gaze error outcome measure RMS-gaze error was also examined with a two-way mixed ANOVA. Where our data departed from normality, main effects were cross checked with a robust mixed ANOVA based on modified M-estimators and bootstrapping (Field et al., 2012). *Post-hoc* analyses (*t*-tests or Wilcoxon signed-rank tests) with Benjamini-Hochberg corrections were used where applicable. Where significant differences were found between conditions (*p* < 0.05), Hedges's *g*_*av*_ effect sizes were calculated as given by Lakens (2013). Common indicative effect thresholds for which include small (0.2), medium (0.5), and large (0.8), respectively.

## 3. Results

### 3.1. Postural sway

**Figure 2:**
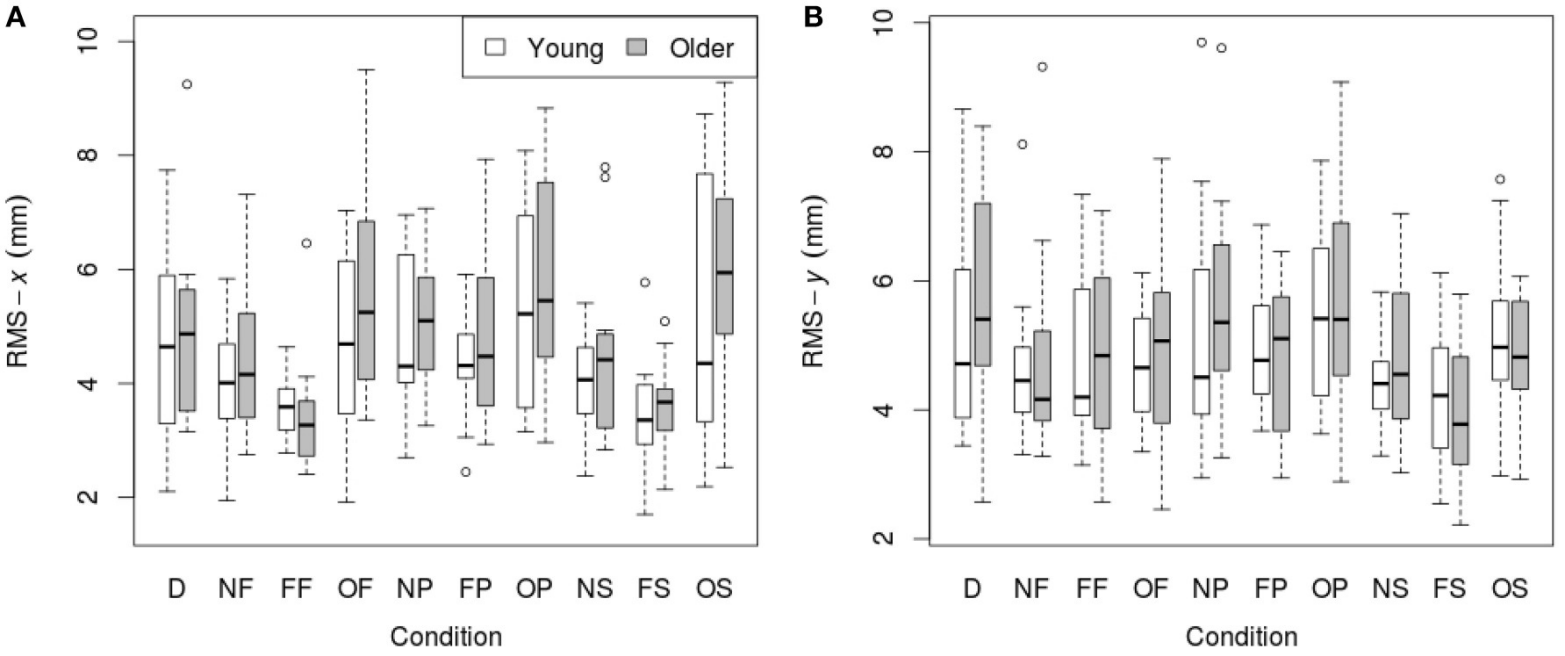
**RMS of COP coordinate timeseries: (A) on the medial/lateral (*x*) axis; (B) on the anterior/posterior (*y*) axis in young (*n* = 12) and older (*n* = 12) participants during different visual scene conditions**. D, dark; N, none; F, fixed; O, oscillating; P, pursuit; S, saccadic. Data are displayed as medians and lower and upper quartiles with Tukey style whiskers (outliers plotted separately).

RMS of the COP coordinate timeseries on the medial/lateral (*x*) and anterior/posterior (*y*) axis for young and older participants are presented in Tables 2, 3 and Figure 2.

**Table 2:** **RMS of COP coordinate timeseries on the medial/lateral (*x*) axis in young (*n* = 12) and older (*n* = 12) participants during different visual scene conditions**.

	**RMS-*x* (mm)**	
**Condition**	**Young**	**Older**
D	4.95 ±1.68	4.70 ± 1.73
NF	4.43 ±1.39	3.99 ±1.11
FF	3.44 ±1.08	3.58 ±0.55
OF	5.69 ±1.89	4.72 ±1.64
NP	5.06 ±1.21	4.85 ±1.43
FP	4.82 ±1.56	4.33 ±0.92
OP	5.81 ±1.96	5.36 ±1.76
NS	4.59 ±1.62	4.01 ±0.85
FS	3.63 ±0.79	3.46 ±1.03
OS	6.32 ±2.31	5.09 ±2.28

D, dark; N, none; F, fixed; O, oscillating; P, pursuit; S, saccadic.

**Table 3:** **RMS of COP coordinate timeseries on the anterior/posterior (*y*) axis in young (*n* = 12) and older (*n* = 12) participants during different visual scene conditions**.

	**RMS-*y* (mm)**	
**Condition**	**Young**	**Older**
D	5.66 ±1.78	5.22 ±1.75
NF	4.79 ±1.70	4.67 ±1.27
FF	5.18 ±2.39	4.78 ±1.30
OF	4.99 ±1.52	4.68 ±0.95
NP	5.89 ±2.15	5.14 ±2.00
FP	4.78 ±1.23	4.94 ±0.89
OP	5.66 ±1.84	5.44 ±1.42
NS	4.80 ±1.29	4.41 ±0.73
FS	3.97 ±1.11	4.26 ±1.12
OS	4.89 ±0.94	5.13 ±1.30

D, dark; N, none; F, fixed; O, oscillating; P, pursuit; S, saccadic.

#### 3.1.1. Medial/lateral (*x*) movement

There was no main effect of age on RMS-*x*. There was a significant main effect of condition on RMS-*x* [*F*_(1, 198)_ = 17.769, *p* < 0.001]. This was confirmed with a robust mixed ANOVA (*p* < 0.001). *Post-hoc* comparisons revealed: (1) A reduction of postural sway with a fixed target in dark (NF) compared to dark alone (D; *p* = 0.032, 12.75%, *g*_*av*_ = 0.40); (2) A reduction of postural sway with a fixed background and a fixed target (FF) compared to dark alone (D; *p* < 0.001, 27.18%, *g*_*av*_ = 0.96), compared to a fixed target in dark (NF; *p* = 0.005, 16.54%, *g*_*av*_ = 0.63), and a reduction of postural sway with a fixed background and saccades (FS) compared to saccades in dark (NS; *p* = 0.001, 17.68%, *g*_*av*_ = 0.66); (3) An increase in postural sway with an oscillating background and a fixed target (OF) compared to a fixed background and a fixed target (FF; *p* < 0.001, 48.20%, *g*_*av*_ = 1.16), an oscillating background and smooth pursuits (OP) compared to a fixed background and smooth pursuits (FP; *p* = 0.001, 22.03%, *g*_*av*_ = 0.62), and an oscillating background and saccades (OS) compared to a fixed background and saccades (FS; *p* < 0.001, 60.91%, *g*_*av*_ = 1.18); (4) An increase in postural sway with smooth pursuits in dark (NP) compared to a fixed target in dark (NF; *p* = 0.038, 17.85%, *g*_*av*_ = 0.57), and smooth pursuits with a fixed background (FP) compared to a fixed target with a fixed background (FF; *p* < 0.001, 30.36%, *g*_*av*_ = 0.95); (5) Saccades did not significantly alter sway compared to a fixed target in any condition; There was no interaction effect between age and condition on RMS-*x*.

#### 3.1.2. Anterior/posterior (*y*) movement

There was no main effect of age on RMS-*y*. There was a significant effect of condition on RMS-*y* [*F*_(1, 198)_ = 4.372, *p* = 0.020]. This was confirmed with a robust mixed ANOVA (*p* < 0.001). *Post-hoc* comparisons revealed: (1) No change in postural sway with a fixed target; (2) No change in postural sway with fixed backgrounds; (3) An increase in postural sway with an oscillating background and saccades (OS) compared to a fixed background and saccades (FS; *p* = 0.008, 21.77%, *g*_*av*_ = 0.78), but no other changes in postural sway with oscillating backgrounds; (4) No change in postural sway with smooth pursuits; (5) No change in postural sway with saccades. There was no interaction effect between age and condition on RMS-*y*.

### 3.2. Gaze error

**Figure 3:**
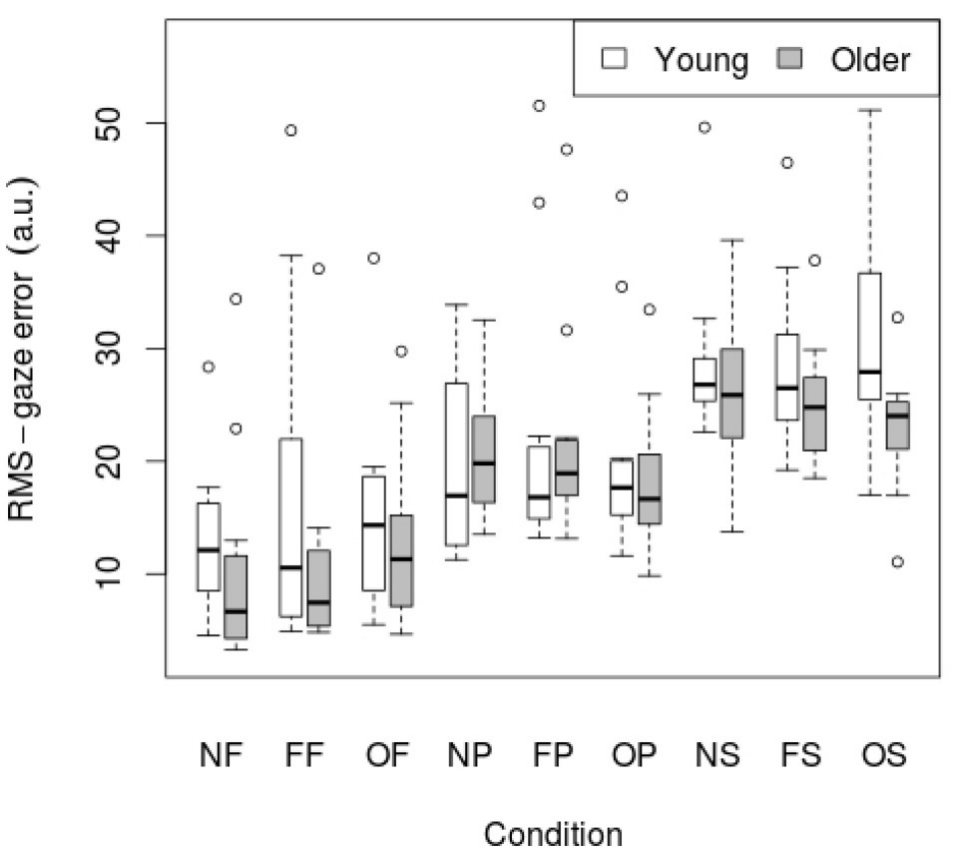
**RMS of gaze subtracted from target position for young (*n* = 12) and older (*n* = 12) participants during different visual scene conditions**. D, dark; N, none; F, fixed; O, oscillating; P, pursuit; S, saccadic. Data are displayed as medians and lower and upper quartiles with Tukey style whiskers (outliers plotted seperately).

RMS of gaze subtracted from target position for young and old participants is presented in Table 4 and Figure 3. There was no significant effect of age on RMS-gaze error. There was a significant effect of condition on RMS-gaze error [*F*_(1, 186)_ = 17.629, *p* < 0.001]. This was confirmed with a robust mixed ANOVA (*p* < 0.001). *Post-hoc* comparisons revealed: (1) No change in gaze error with fixed or oscillating backgrounds; (2) An increase in gaze error with smooth pursuits in dark (NP) compared to a fixed target in dark (NF; *p* < 0.001, 74.37% *g*_*av*_ = 1.13), smooth pursuits with a fixed background (FP) compared to a fixed target with a fixed background (FF; *p* = 0.007, 57.4% *g*_*av*_ = 0.67), and smooth pursuits with an oscillating background (OP) compared to a fixed target with an oscillating background (OF; *p* = 0.001, 38.61%, *g*_*av*_ = 0.64); (3) An increase in gaze error with saccades in dark (NS) compared to smooth pursuits in dark (NP; *p* = 0.001, 34.22%, *g*_*av*_ = 0.98), saccades with a fixed background (FS) compared to smooth pursuits with a fixed background (FP; *p* = 0.016, 23.22%, *g*_*av*_ = 0.55), and saccades with an oscillating background (OS) compared to smooth pursuits with an oscillating background (OP; *p* = 0.001, 38.63%, *g*_*av*_ = 0.87); There was no interaction effect between age and condition on RMS-gaze error.

**Table 4:** **RMS of gaze subtracted from target position (in arbitrary units) for young (*n* = 12) and older (*n* = 12) participants during different visual scene conditions**.

	**RMS-gaze error (a.u.)**	
**Condition**	**Young**	**Older**
NF	10.33 ± 9.35	13.06 ± 6.32
FF	10.63 ± 8.97	16.98 ± 14.23
OF	12.90 ± 7.76	14.91 ± 8.77
NP	20.85 ± 5.82	19.94 ± 8.13
FP	21.73 ± 9.38	21.74 ± 12.34
OP	18.11 ± 6.61	20.44 ± 9.44
NS	25.96 ± 6.69	28.78 ± 7.09
FS	25.16 ± 5.40	28.39 ± 7.50
OS	22.87 ± 5.29	30.58 ± 9.55

D, dark; N, none; F, fixed; O, oscillating, P, pursuit; S, saccadic.

## 4. Discussion

The present work aimed to assess the effects of eye movements on balance in young and older individuals. We took a novel approach by assessing postural sway during three primary occulomotor behaviors with different forms of retinal flow, whilst simultaneously assessing gaze accuracy. Alterations of posture with different visual conditions were found predominantly on the medial/lateral (*x*) axis, with fixed stimuli having a stabilizing effect, and oscillating backgrounds and smooth pursuits having a destabilizing effect. There were no differences between age groups for any of the posture and gaze measures. The underpinning mechanisms and potential causes are discussed.

### 4.1. Visual fixation of a stationary target

In support of extraocular postural control, or the ability of the CNS to interpret eye movement signals to gain positional information (Guerraz and Bronstein, 2008), we found a decrease of body sway when visually fixating a stationary target in dark. Two lines of reasoning have been discussed to explain this phenomenon; the inflow and outflow hypotheses. The former suggests that proprioceptors located in the extraocular muscles provide information about the magnitude of eye movements, which can be interpreted for estimates of body shifts during postural sway. This can only occur after eye movements have been initiated. The latter suggests such information can be gained from a copy of the motor command used to signal eye movements, or neural outflow used by the CNS to maintain visual consistency, and thus the magnitude of the eye movements may be anticipated in a feed forward manner.

Since there were no changes in postural sway with age in this condition, it seems likely the older participants were able to perceive head motion relative to the target as effectively as the young group. There were also no changes in gaze errors with age, which indicates a similar reduction of retinal flow for both young and older. Therefore, the extraocular factors involved in the control of posture might have been preserved. Because maintaining gaze on a fixed target requires compensatory eye movements, initiated in part by the VOR, the present findings also suggest that the elders had no substantial VOR deficits, which lends support to a recent study indicating such declines are limited to individuals aged 80 years and over (Li et al., 2015). To this point, our suggestion that age-related declines in VOR may affect extraocular balance control seems not to have occurred in our participants. Future research should seek to examine extraocular postural control mechanisms in populations with known VOR deficits.

### 4.2. Fixed and oscillating backgrounds

The addition of fixed backgrounds attenuated postural sway during all eye movements apart from smooth pursuits (discussed below). This reflects integration of the static visual field, and thus retinal flow, into the postural control system, allowing for more accurate visual estimates of body position (Glasauer et al., 2005; Laurens et al., 2010). The magnitude of gaze errors did not change, suggesting the participants were not distracted from the visual target.

Oscillating backgrounds generating horizontally translational retinal flow absent of parallax cues had a destabilizing effect during all eye movements. Previous work examined coupling of postural sway to stimulus motion with frequency response ratios (Logan et al., 2010). Strong coupling typically occurs at frequencies below 0.2 Hz, which is believed to be a result of the CNS misinterpreting external-motion for self-motion and initiating incorrect postural responses. At higher frequencies (>0.3 Hz), coupling is largely diminished (Guerraz and Bronstein, 2008). This is logical, considering if coupling were to remain, loss of balance might ensue. Since oscillation of the background in the present study was 0.33 Hz, and the participants did not lose their balance, it is likely there was a weak or no coupling of body sway with the background, probably through distinguishing between retinal flow caused by self-motion, and retinal flow caused by external-motion (DeAngelis and Angelaki, 2012). Vestibular and proprioceptive signals may be of particular importance in such a process, since they provide independent sources of information about head and body position in space (DeAngelis and Angelaki, 2012). Notwithstanding this, there were still increases in postural sway. This may be attributed to more challenging integration of the non-static retinal flow. In effect, it was likely harder to make visual estimates of body position against the dynamic background visual field. Interestingly, this occurred even with the stationary fixed target in the center of the field of vision, which supports the theory that the central area of the retina at which the fixed target would have been located is associated more with object recognition (Guerraz and Bronstein, 2008), and the peripheral visual field in which the oscillating background would be located is more dominant in control of posture in moving visual fields (Piponnier et al., 2009). In this respect, it seems the effect of the retinal flow was stronger than potential extraocular factors which might have been at play. There were no differences in gaze errors when oscillating backgrounds were added, suggesting again that the participants were not distracted from the target.

We found no differences between age groups for static or oscillating backgrounds. This was surprising as older individuals typically demonstrate greater body sway when standing in both stable visual information rich environments, such as a lit room, (Prieto et al., 1996) and in oscillating visual fields (Wade et al., 1995; Sundermier et al., 1996; Borger et al., 1998). We normalized the data to body height and body mass which have been shown to be determinants of postural sway in females during feet together stance (Kim et al., 2010) but were still unable to find any changes. This suggests that the older participants integrated all of the visual information for postural control as effectively as the young group, including determining body shifts from static and dynamic visual fields, and solving the external-motion from self-motion separation issue. We also found no differences in gaze errors between age groups with the addition of fixed or oscillating background information. Previous findings have suggested that elders may be more distracted by background motion, possibly related to a reduction in GABA-mediated inhibition, and this may have consequences for discriminating motion of moving objects from their backgrounds (Tadin and Blake, 2005). The present results do not support this idea.

### 4.3. Smooth pursuits

Smooth pursuits increased postural sway in the absence of retinal flow. We suggested above that eye movement signals were used to infer body position during fixation of a stable target with no background information (extraocular balance control). An increase in task complexity during smooth pursuits may complicate such extraocular signals, which in turn may have caused the increase in postural sway. The neural basis of these findings goes beyond the scope of this investigation, but might be related to the factors previously outlined.

Tracking a moving target over a fixed background also increased postural sway, yet we predicted the static visual field would have a stabilizing effect. One can argue that preserving stability of a given visual field on the retina is important for accurate measurement of postural shifts (Schulmann et al., 1987). During smooth pursuits, however, the image of the visual target may appear stable on the fovea (Thier and Ilg, 2005), but the background visual field shifts on the retina in the opposite direction to the target movement (Schulmann et al., 1987). This would generate similar retinal flow patterns to an oscillating background visual field, which may in turn lead to more challenging conditions for estimation of body position. Such results also support the notion that whilst smooth pursuits are good at maintaining the image of an object on the fovea, subserving a central analytical function, they are not efficient regarding spatial orientation, due to apparent motion of the background in the peripheral visual field (Schulmann et al., 1987).

In the previous experiments, the addition of a fixed background reduced the effect of the moving target on postural sway (Glasauer et al., 2005; Laurens et al., 2010). The differences between these and the present findings could be related to the nature of the stimulus movement. In the previous investigations, stimulus trajectory consisted of either horizontal, or vertical oscillations, which may have been easy to predict. In the present experiment, target movement was random on the vertical, horizontal and diagonal axis during each condition reflecting more unpredictability, more complex movement of the background visual information, and more complex extraocular signals. Thus, integration of retinal flow into the postural control system might have been more challenging, and this reduced the effect of an otherwise stabilizing visual anchor.

Our findings contrast with Rodrigues et al. (2015) who found a reduction of body sway during smooth pursuits. A potential cause lies with more challenging foot placement strategies used in the present investigation (and in Laurens et al., 2010 and Glasauer et al., 2005). Rodrigues et al. (2015) suggested postural sway was attenuated to gain more accurate gaze control during normal stance. When standing with feet together, or on foam/semi tandem stance in the previous experiments, such attenuation did not occur. It seems likely, therefore, that stance position dictates the outcome of postural response during smooth pursuits in the presence of stable visual background information. As Rodrigues et al. (2015) did not assess smooth pursuit movements independent of background visual information, it cannot be inferred whether stance would have any impact in such conditions.

Surprisingly, there were no differences between age groups for balance during smooth pursuits in any condition. It is thus possible that the older participants processed the potentially more complex extraocular signals, and dynamic retinal flow for postural control as efficiently as the young group. We also found no differences between age groups for gaze errors. This contradicts previous results showing age-related declines in smooth pursuit accuracy (Sharpe and Sylvester, 1978; Spooner et al., 1980; Moschner and Baloh, 1994; Ross et al., 1999; Knox et al., 2005). It may be the Tobii I-VT fixation filter we used to process the raw gaze data being a velocity-threshold identifier was not sufficiently accurate to discern small changes between the age groups which would require finer grained gaze data analysis such as that previously used Paquette and Fung (2011). With that said, a recent study found no difference between smooth pursuit parameters of young and older adults tracking targets in an ecologically valid environment (Dowiasch et al., 2015). We cannot ultimately say for sure which previous results would appropriately describe our participants. However, our previous suggestion that a decline in the accuracy of the smooth pursuit system with age may affect extraocular control of balance is incorrect, at least in our participants.

### 4.4. Saccades

We found no changes in postural sway during saccades compared to fixating a stable target in the absence of a visual background. Since in both conditions, the target was the predominant source of visual information, one must assume a similarity in the way it was used for postural control. This may be explained by the frequency of the target movement (0.33 Hz). Each saccadic shift of the target was completed at the projector refresh rate, in the order of sub 20 ms. Consequently, the target remained at the center position, or at 6° of visual angle at any given trajectory, for close to 1.5 s on each half oscillation. Since a saccadic shift of the human eye also with a displacement of 6° can be completed in around 40.6 ms (Abrams et al., 1989), gaze would have been fixated on a static target for relatively long periods during the saccadic trials aside from corrective saccades due to gaze errors. This suggests that similar to fixating a static target in dark, extraocular factors were involved in balance control. Future investigations should examine such extraocular contributions, during saccades with a range of movement frequencies.

The addition of a fixed background did attenuate postural sway further. As saccades aim to depict the visual environment as stable, e.g., to connect pre- and post-saccadic views, and gaze would have been fixated in the same position for relatively long periods, as above, the CNS might gain better estimates of head position from the background visual field in this condition (Schulmann et al., 1987), which seems to have occurred in our experiment regardless of changes in eye orientation.

The present findings do not align with previous data showing improvements in upright stability during saccades (Rodrigues et al., 2013, 2015; Aguiar et al., 2015). Stance position was the same as in Aguiar et al. (2015) and Rodrigues et al. (2013) and thus can be excluded as a causal factor. In these previous investigations, the authors suggested that postural sway was modulated to afford more accurate gaze shifts, since they found more sway attenuation at higher frequency saccades (1.1 Hz compared to 0.5 Hz). The frequency of saccades in the present investigation was lower at 0.33 Hz, and may not have required the same magnitude of postural sway attenuation.

We additionally found no differences in postural sway or gaze error between age groups during saccades. Therefore, the older participants may have been visually fixated on the target for similar time scales as the young group, suggesting a similar amount of positional information was interpreted, either extraocular or from retinal flow. Although it is possible that we failed to detect small effects of age on saccadic accuracy, such as longer onset latencies, or more saccades to reach the target (Paquette and Fung, 2011), this certainly had no effect on the postural outcomes.

Another possible explanation as to why we found no differences for postural sway with age during saccades and smooth pursuits relates to rigidity. Melzer et al. (2001) showed that when performing a dual task whilst stood with their feet together, elders reduced their body sway by increasing muscle activity in the tibialis anterior and soleus muscles. This coactivation about the ankle was thought to be a consequence of a threat to postural stability. Other findings from older individuals also point toward increases in muscle coactivation during standing, which may be a mechanism to compensate for natural age-related declines in the postural control system (Nagai et al., 2011). Such a mechanism has indeed been suggested to occur during saccadic eye movements (Aguiar et al., 2015). In the present study, the older participants may have been more challenged in terms of central integration of visual cues for postural control, and subsequently adopted a more rigid postural response through muscle coactivation, but this was not detected through measures of postural sway alone. Simultaneous assessment of muscle activity would be needed to confirm or reject this idea.

The present findings demonstrate the effects of eye movements on postural control in young and older females. In younger males and females, similar effects have previously been demonstrated (Glasauer et al., 2005; Laurens et al., 2010). In older males, we hypothesize that our findings would be replicated, since a previous study which manipulated visual parameters, in elders, was unable to detect significant gender differences in postural sway during quiet stance (Wolfson et al., 1994).

### 4.5. Axis effects

The only change in posture on the anterior/posterior (*y*) axis was found with the addition of an oscillating background, whilst all other changes were found on the medial/lateral (*x*) axis. This indicates more stability on the anterior/posterior (*y*) axis compared to the medial/lateral (*x*) axis overall, which likely results from a reduced base of support on the medial/lateral (*x*) axis during feet together stance compared to normal stance. With that said, we did not utilize anterior/posterior (*y*) translations of the visual background during the eye movements to generate expansion and contraction retinal deformation patterns. Such conditions may have caused greater instability on this particular axis during eye movements, similar to changes in postural sway previously shown by Jeka et al. (2008). This is a recommendation for future experiments.

### 4.6. Method consideration

With regard to previous studies investigating postural sway during eye movements, the participants were instructed to focus on the visual stimuli, but not directly examined as to whether they did so. The present results suggest that such instruction is appropriate and participants are able to remain fixated on the target, aside from natural gaze errors. Therefore, we suggest this set-up should continue being used for assessment of postural sway and eye movements during quiet stance.

### 4.7. Conclusion

The present investigation supports growing evidence that eye movements interact with the postural control system in humans, which could have important implications for practitioners and researchers working with a variety of populations. Extraocular components have been shown to contribute to postural control in a number of laboratory conditions. Thus, if extraocular balance control is impeded in individuals with substantial declines in VOR and/or visual proprioceptive function, discerning the relative contribution of extraocular and retinal mechanisms to balance control in an ecologically valid environment and during different eye movements would be an important step in developing a targeted training intervention. Moreover, since we and other studies found increases of postural sway during smooth pursuits in more challenging stance positions, stability whilst tracking moving targets may also be affected during locomotion or perturbed stance. This could place populations less able to correct postural disturbances, including elders, at a greater risk of falls. Should such individuals be instructed to refrain from observing moving objects, thus suppressing visual tracking, and only utilize static fixations and saccades which maintain or improve stability to scan their environment? Or perhaps training programs should focus on improving postural control during smooth pursuit eye movements in a variety of conditions. Some of these points were first raised by Schulmann et al. (1987). Here, we suggest further research is still needed, and should also take account of extraocular factors. With that said, in the present context, our older participants were able to match the younger group's postural and visual performances. This may be said on the cognitive level (sensory integration of visual cues to the postural control system), and on the physical functioning level (musculoskeletal responses to maintain upright stabilty). How this translates to more dynamic situations such as locomotion, and with different populations, now remain the topics of interest.

## Author contributions

NT conceived the investigation, led the data collection and analysis and interpretation of the results, and drafted the first manuscript. TB, TD, and SD contributed to all aspects of the investigation, including methodological design, data collection, and analysis, interpretation of the results, and revision of the manuscript for important intellectual content. All authors approved the final version of the manuscript and agree to be accountable for all aspects of the work.

### Conflict of interest statement

The authors declare that the research was conducted in the absence of any commercial or financial relationships that could be construed as a potential conflict of interest.
